# Perceptions of Family Physician Trainees and Trainers Regarding the Usefulness of a Virtual Community of Practice

**DOI:** 10.2196/jmir.2555

**Published:** 2013-05-10

**Authors:** Stephen Barnett, Sandra C Jones, Sue Bennett, Don Iverson, Andrew Bonney

**Affiliations:** ^1^General Practice Academic UnitGraduate School of MedicineUniversity of WollongongWollongongAustralia; ^2^Centre for Health InitiativesUniversity of WollongongWollongongAustralia; ^3^Faculty of EducationUniversity of WollongongWollongongAustralia; ^4^Health and Behavioural SciencesUniversity of WollongongWollongongAustralia

**Keywords:** community of practice, virtual community of practice, general practice, family physician, training, education, medical graduate, social media

## Abstract

**Background:**

Training for Australian general practice, or family medicine, can be isolating, with registrars (residents or trainees) moving between rural and urban environments, and between hospital and community clinic posts. Virtual communities of practice (VCoPs), groups of people sharing knowledge about their domain of practice online and face-to-face, may have a role in overcoming the isolation associated with general practice training.

**Objective:**

This study explored whether Australian general practice registrars and their supervisors (trainers) would be able to use, and would be interested in using, a VCoP in the form of a private online network for work and training purposes. It also sought to understand the facilitators and barriers to intention to use such a community, and considers whether any of these factors may be modifiable.

**Methods:**

A survey was developed assessing computer, Internet, and social media access and usage, confidence, perceived usefulness, and barriers, facilitators, and intentions to use a private online network for training purposes. The survey was sent by email link to all 139 registrars and 224 supervisors in one of Australia’s 17 general practice training regions. Complete and usable responses were received from 131 participants (response rate=0.4).

**Results:**

Most respondents had access to broadband at home (125/131, 95.4%) and at work (130/131, 99.2%). Registrars were more likely to spend more than 2 hours on the Internet (*P*=.03), and to use social media sites for nonwork purposes (*P*=.01). On a 5-point Likert scale, confidence was high (mean 3.93, SD 0.63) and was negatively associated with higher age (*P*=.04), but not associated with training stage. Social media confidence was lower, with registrars more confident than supervisors for almost all social media activities. On a 5-point Likert scale, overall usefulness was scored positively (n=123, mean 3.63, SD 0.74), and was not significantly associated with age or training level. The main concerns of respondents were worries about privacy (registrar: 61/81, 75.3%; supervisor: 30/50, 60.0%) and insufficient time (registrar: 41/81, 50.6%; supervisor: 36/50, 72.0%). Using a multivariate generalized linear regression model, training stage and perceived usefulness were positively predictive, and concerns about privacy and time were negatively predictive of intention to use a private online network.

**Conclusions:**

General practice registrars and supervisors are interested in using a private online network, or VCoP, for work and training purposes. Important considerations are the extent to which concerns such as privacy and usefulness may be overcome by training and support to offset some other concerns, such as time barriers. Participants at an early stage in their training are more receptive to using an online network. More senior registrars and supervisors may benefit from more training and promotion of the online network to improve their receptiveness.

## Introduction

Training for general practice, or family medicine, in Australia is a postgraduate specialty program. After graduation from medical school, doctors spend a minimum of 1 year in the hospital system. To become a general practitioner, they must join a 3-year general practice training program run by one of 17 regional training providers across Australia. This program consists of 1 hospital year and 2 supervised general practice years. During these 3 years, trainees are required to work in a number of different rural and urban general practice locations, with at least 6 months located in a rural area. These locations are often small practices with a limited number of medical colleagues on-site, in contrast to the large hospitals with many colleagues that characterize early medical training.

As a result of these features, general practice training can be isolating [1], resulting in decreased knowledge sharing [2], and can affect career choices [3], including lowering intention to work in rural areas [1]. This has implications for the quality of training, standard of the primary care workforce, and retention of a rural general practice workforce.

The types of isolation experienced can be categorized as structural, personal, and professional [1]. Structural isolation refers to smaller practices with closed consulting rooms and occurs across urban and rural sites [1]. Social isolation, which can be described as a kind of loneliness [4], is more common in rural placements [1,5]. Professional isolation results from a lack of clinical support and is also potentially a greater problem in rural areas [1]. Professional isolation is linked to barriers to knowledge sharing, with reduced tacit knowledge exchange, and networking opportunities [2]. Problems with training, including all 3 types of isolation, are associated with a decreased intention to work in rural or regional areas [1].

The general practice workforce in Australia is under pressure [6], particularly in rural areas [7]. Given that isolation can lead to a lower intention to practice in rural and regional areas, it is important to try to overcome isolation to maintain a sustainable general practice workforce.

A recent literature review proposed a role for virtual communities of practice (VCoP) in overcoming isolation, particularly professional isolation, through improved knowledge sharing [8]. The literature review built on an accepted business VCoP framework [9], proposing a framework for VCoPs in health. Communities of practice (CoPs) are “groups of people who share a concern or a passion for something they do and learn how to do it better as they interact regularly” [10]. These groups build shared resources that maintain ways of working, standards, and values within the community [11,12]. As technology has progressed, collaboration is being facilitated by social media tools [13-15] resulting in a blending of face-to-face and virtual communities of practice [16,17]. This differs from a simple virtual community that is fluid and without formal boundaries or membership [18] and, most importantly, may be purely based on a shared interest, such as movie trivia, rather than a shared practice. Probst and Borzillo [9] have developed a framework for CoPs implementation on the basis of 57 face-to-face and virtual CoPs in large companies such as IBM and Siemens. Barnett et al [8] have refined this for the health sector after a comprehensive review of the health literature and suggested a role for VCoPs, in the form of online private networks, in overcoming isolation through improved interaction with colleagues and knowledge sharing.

This study explored whether Australian general practice trainees and their supervisors would be able to use, and would be interested in using, a VCoP of this type for work and training purposes. It also sought to understand the facilitators and barriers to intention to use, such as community, and considered whether any of these factors could be modified.

## Methods

### Participants

The sampling frame for the current study included all general practice trainees and supervisors in a large regional training provider in Australia in May 2010. In ascending order, the training levels are basic registrar, advanced registrar, subsequent registrar, supervisor, and educator. The training provider, Coast City Country General Practice Training (CCCGPT), provides general practice training across a wide geographic area, including the urban centers of Canberra in the Australian Capital Territory and Wollongong in New South Wales, alongside large regional and small rural centers spread across approximately 160,000 square kilometers.

Surveys were sent to all trainers and trainees on the CCCGPT database via an email link to SurveyMonkey [19], a Web-based survey creation tool. A participant information sheet was provided. Surveys were sent to the total sampling frame of 363 people, which included 139 registrars and 224 supervisors. A total of 146 completed surveys were returned (40.2%); 15 participants were removed for reasons such as not completing at least half of the survey (n=10), not completing demographic data (n=3), and not ticking the consent box on the survey (n=2). This left 131 (36.1%) for analysis.

Ethics approval was obtained from the University Human Research Ethics Committee.

### Questionnaire

There is a lack of literature on VCoPs in general practice training [8]; therefore, the survey was developed by the authors to assess computer, Internet, and social media access and usage, confidence, perceived usefulness, intentions to use, and barriers to use for training purposes.

The instrument was piloted among a group of general practitioners, general practice trainees, and health researchers. Afterwards, a group discussion among pilot participants led to the amendment of wording and several response options alterations, to improve clarity and better reflect GP work.

The final survey consisted of 26 questions, including categorical and Likert response items (see Table 1). Specifically, the questions covered demographics (questions 1-5), computer and Internet access and usage (questions 6-9), computer and social media confidence (questions 10 and 11), social networking usage (questions 12-21), social media usefulness (questions 22 and 27), barriers to use (questions 23 and 24), and intention to use social media for training purposes (questions 25 and 26).

### Statistical Analysis

Data were analyzed using SPSS version 19 (IBM Corp, Armonk, NY, USA). Respondents were categorized as registrar or supervisor for comparisons between groups. The *t* test and chi-square test were used to determine differences between responses based on rurality, gender, age, and training level. Paired-samples *t* tests were used to compare means of scale data, such as intention to use a private social network for work purposes and intention to use an open social network for work purposes. Independent-sample *t* tests were used to compare categorical and scale data, such as computer confidence, and for the analysis involving all categories of training level. The chi-square test was used to compare differences between categorical data, such as rurality and training level. All statistical comparisons were 2-tailed and statistical significance was set at *P*<.05.

Factor analysis using varimax rotation was used to determine which Likert items grouped naturally in questions with multiple Likert items for constructs such as computer confidence (questions 10 and 11) and usefulness (question 22). Factors were included if their eigenvalues were >1.0. The Cronbach alpha test for reliability was used to determine the degree of agreement between the Likert items. Cronbach alpha was >.8 for both items, higher than the recommended threshold of .70.

A confidence scale was constructed using all items from questions 10 and 11; the summated data were used as an independent variable in further analysis. The Pearson product moment correlation (*r*) was used to determine agreement between variables, such as confidence and intention to use a private network for training purposes. The multivariate associations of independent variables, such as confidence and training level, with the dependent variable of intention to use a private network for training purposes were examined using multivariate general linear regression modeling.

**Table 1:** Survey content and question type.

Question content	Question type	Question number (categorical options or Likert items)
Demographic	Categorical	1 (2), 2 (2),3 (1), 4 (2), 5 (2)
Access and usage	Categorical	6 (2), 7 (2), 8 (6), 9 (7)
Confidence	Likert items	10 (4), 11 (7)
Social networking usage	Categorical	12 (2), 13 (9), 14 (11), 15 (2), 16 (9),17 (2), 18 (1), 19 (2), 20 (5), 21 (8)
Usefulness	Likert items	22 (14)
Usefulness	Categorical	27 (6)
Barriers	Categorical	23 (8), 24 (8)
Intention to use	Likert items	25 (2), 26 (2)

## Results

### Characteristics of the Survey Population

Of the 131 respondents, gender was evenly split (males: 66/131, 50.4%; females: 65/131, 49.6%). Registrars accounted for 61.8% (81/131) of respondents and the remainder were supervisors. The response rate among trainees was higher than supervisors (registrar: 81/139, 58%; supervisor: 50/224, 22%). The mean age of the sample was 41.5 years (range 23-66 years, SD 10.369), with a significant difference between ages of trainees and supervisors (trainees: mean 35.9, SD 7.21; supervisors mean 51.0, SD 7.21, *P*<.001).

Over half (75/131) of respondents were from rural settings, whereas the remainder worked in a general (nonrural) setting, with no significant differences between training stage and rurality or age and rurality.

### Access and Usage

Almost all general practice trainees and supervisors had access to broadband Internet at home (125/131, 95.4%) and at work (130/131, 99.2%). However, usage was found to be significantly different between registrars and supervisors, with 20.0% (10/50) of supervisors compared to 33.3% (27/81) of registrars spending more than 2 hours per day on the Internet (*P*=.03). Internet usage of greater or less than 2 hours per day was not significantly associated with age (*P*=.17)

Registrars were significantly more likely to use social networking sites for nonwork purposes (registrars: 41/81, 50.6%; supervisors: 14/50, 28%, *P*=.01), and higher usage was associated with lower age (*P*<.001). Both registrars and supervisors were unlikely to use social networking sites for work purposes (registrars: 13/81, 16.0%; supervisors: 4/50, 8.0%) and there was no statistically significant difference between the groups.

Out of all online social media activities, registrars and supervisors were most likely to watch online videos (registrars: 63/81, 77.8%; supervisors: 27/50, 54.0%), followed by reading discussions (registrars: 53/81, 65.4%; supervisors: 25/50, 50.0%). They were least likely to construct a wiki (registrars: 3/81, 3.7%; supervisors: 0/50, 0.0%). Video watching was significantly correlated with age, with younger users watching more video (*P*=.001) and registrars watching more video than supervisors (*P*=.004). Reading online discussions was not significantly different between registrars and supervisors and was not associated with age.

### Confidence

Factor analysis was performed on the 4 general computer confidence items, revealing only 1 factor, which was labeled *computer confidence*. The factor analysis was reliable (Cronbach alpha=.82) and valid (eigenvalue=2.66). Overall confidence was high (n=131, mean 3.93, SD 0.63) and confidence was negatively associated with age (*r*= –0.18, *P*=.04), but not significantly associated with being a registrar or a supervisor.

Confidence using discussion boards, wikis, blogs, online communities, chat, online video, and Twitter was assessed on a 5-point Likert scale for each of the 7 items. Confidence among supervisors was low to moderate, from a mean of 2.32 (SD 0.91) to a mean of 2.98 (SD 1.29), and was significantly lower than among registrars for all applications except Twitter, which was low for both groups (see Table 2).

Factor analysis was performed on the 7 social media confidence items, revealing only 1 factor which was labeled *social media confidence*. The factor analysis was reliable (Cronbach alpha=.93) and valid (eigenvalue=5.0). Social media tool confidence overall was moderate (n=131, mean 3.03, SD 0.99) and was negatively associated with age (*r*=–0.38, *P*<.001) and training level (*P*<.001), with younger respondents and registrars more likely to be confident with social media tools.

Cronbach alpha for the items in the confidence scale including all 11 items was .92. The inter-item correlations ranged between 0.21 and 0.78 indicating that there were no redundant items.

### Usefulness

Using a 5-point Likert scale, 13 items were asked regarding perceived usefulness of social networks, regardless of whether the respondent currently used social networks, for aspects such as training purposes, keeping in touch with other trainees, job networking, and social support (Table 3).

The question “keeping in touch with other registrars” was the only item to show a significant difference between registrars and supervisors (*P*=.002). On review of the result, it was decided that the question was confusing because supervisors were being asked to value the usefulness of keeping in touch with other registrars, for which they have little need, as opposed to keeping in touch with other supervisors. Because of the confusing nature of the question, it was discarded from the subsequent factor analysis. Factor analysis of the remaining 12 items revealed a single factor (Cronbach alpha=.96; eigenvalue= 8.3) labeled *usefulness*. Overall usefulness was scored positively (n=123, mean 3.63, SD 0.74), and was not significantly associated with age or training level. Usefulness was not significantly correlated with computer confidence, but was significantly correlated with social media tool confidence (*r*=0.27, *P*=.02).

### Barriers to Use

A number of barriers to using social networks for work were described. The main concerns were worries about privacy (registrar: 61/81, 75.3%; supervisor 30/50, 60.0%) and insufficient time (registrar: 41/81, 50.6%; supervisor: 36/50, 72.0%; see Table 4). Factor analysis was not performed as these barriers were categorical questions.

### Intention to Use

An important aim of the survey was to assess whether doctors would use a social network for training purposes. Respondents were asked whether they would use a private network or an open network, such as Facebook, for work purposes or social purposes.

Respondents differed in their intentions to use private as compared with open networks. All respondents were significantly more likely to use a private network for work purposes compared to using an open network for work purposes (*P*<.001). On subgroup analysis, both registrars and supervisors were more likely to use a private network for work purposes than an open network (*P*<.001), but registrars were more likely to use a private network for work purposes than supervisors (*P*<.001). Both registrars and supervisors were equally likely to use an open or private network for social purposes (Table 5).

To investigate which factors had an independently predictive value for the outcome “I would use a private network for work and training purposes,” a multivariate generalized linear regression model was developed using private work as the dependent variable. To inform this model, multiple correlations and *t* tests were performed to identify individual factors that correlated with the intention to use a private network for work and training purposes (Table 6). These factors were then entered into the regression model as independent factors.

In the initial model, age was not independently predictive, whereas training level was predictive. Given that training level is related to age, the subcategories of training status were analyzed in the model.

The final model was significant (*R* ^*2*^=.365). In the final model, controlling for other factors, training level was an independently significant predictor of intention to use a private network for work and training. The beta coefficient fell as training level rose, showing the most significant predictor was early training stage, declining as registrars progressed through training. Concerns about privacy and time were negatively predictive, whereas security concerns were nonsignificant. Usefulness was independently predictive of use of a private network for work and training purposes. Confidence was not statistically significant (*P*=.06; see Table 7).

**Table 2:** Means and standard deviations for confidence using Internet-based applications and services.

Item and group^a^		n	Mean	SD	*t*_129_	*P*	95% CI	
							LL	UL
**Discussion forums**					2.05	.04	0.01	0.82
	Registrars	81	3.40	1.02				
	Supervisors	50	2.98	1.29				
**Wikis**					4.21	<.001	0.44	1.21
	Registrars	81	3.22	1.07				
	Supervisors	50	2.60	1.11				
**Blogs**					2.68	.008	0.14	0.91
	Registrars	81	3.12	1.02				
	Supervisors	50	2.60	1.20				
**Online communities (eg, Facebook)**					4.17	<.001	0.46	1.30
	Registrars	81	3.48	1.22				
	Supervisors	50	2.60	1.23				
**Online chat/instant messaging**					3.98	<.001	0.40	1.27
	Registrars	81	3.46	1.22				
	Supervisors	50	2.62	1.24				
**Online video**					3.60	<.001	0.34	1.13
	Registrars	81	3.69	1.01				
	Supervisors	50	2.96	1.26				
**Twitter**					1.32	.19	–0.12	0.59
	Registrars	81	2.56	1.04				
	Supervisors	50	2.32	0.91				

^a^ Likert scale: 1=strongly disagree, 2=disagree, 3=neither agree nor disagree, 4=agree, 5=strongly agree.

**Table 3:** Responses of registrars and supervisors about the usefulness of social networks.

Item and group^a^		n	Mean	SD
**Training purposes**				
	Registrars	80	3.60	1.01
	Supervisors	49	3.43	0.82
**Keeping in touch with other registrars**				
	Registrars	80	4.11	0.83
	Supervisors	48	3.69	0.55
**An extra way of interacting with current supervisors**				
	Registrars	79	3.37	1.12
	Supervisors	49	3.61	0.76
**A way of interacting with previous supervisors/other clinical mentors**				
	Registrars	79	3.61	0.93
	Supervisors	49	3.63	0.57
**Job networking**				
	Registrars	80	3.61	0.95
	Supervisors	49	3.59	0.65
**Staying in touch with people**				
	Registrars	79	3.96	0.86
	Supervisors	49	3.78	0.65
**Social support from peers**				
	Registrars	80	3.60	0.99
	Supervisors	49	3.63	0.67
**Professional support from peers**				
	Registrars	80	3.60	0.99
	Supervisors	49	3.63	0.10
**Professional support from supervisors**				
	Registrars	80	3.40	1.06
	Supervisors	49	3.63	0.71
**A knowledge resource for solving clinical problems with the help of other clinicians**				
	Registrars	79	3.58	1.01
	Supervisors	49	3.47	0.82
**A way of sharing useful resources with colleagues**				
	Registrars	80	3.81	0.94
	Supervisors	49	3.63	0.67
**A forum for expressing or hearing opinions on clinical and political topics**				
	Registrars	80	3.64	0.98
	Supervisors	49	3.65	0.72
**A resource of useful learning tools (eg, video tutorials)**				
	Registrars	80	3.86	0.92
	Supervisors	48	3.65	0.76
**Other**				
	Registrars	24	3.13	0.68
	Supervisors	14	3.50	0.76

^a^ Likert scale: 1=strongly disagree, 2=disagree, 3=neither agree nor disagree, 4=agree, 5=strongly disagree.

**Table 4:** Perceived difficulties in using online social networks for professional purposes.

Difficulty	Registrars, n (%) n=81	Supervisors, n (%) n=50
Worried about privacy	61 (75.3)	30 (60.0)
Insufficient time	41 (50.6)	36 (72.0)
Worried about security	39 (48.1)	19 (38.0)
Not sure how to use them	22 (27.2)	20 (40.0)
Not interested	12 (14.8)	17 (34.0)
Technical Issues	23 (28.4)	9 (18.0)
Lack of other colleagues known to use them	27 (33.3)	22 (44.0)
Other	4 (4.9)	4 (8.0)

**Table 5:** Private versus open network usage among registrars and supervisors.

Item and group		Open mean (SD)	Private mean (SD)	*P*
**Work**				
	All	2.09 (0.97)	3.57 (0.93)	<.001
	Registrars	2.2 (0.99)	3.85 (0.77)	<.001
	Supervisors	1.9 (0.90)	3.16 (0.97)	<.001
**Social purposes**				
	Registrars	3.21 (1.30)	3.19 (1.10)	.85
	Supervisors	2.40 (1.35)	2.62 (1.05)	.25

**Table 6:** Factors correlated with the intention to use a private network for work or training purposes.

Factor	Significance (*P*)
Training level: supervisor or registrar	<.001
Rural versus urban	.42
Age	.01
Confidence (computer + social)	.03
Usefulness	.03
Concern about privacy	.11
Concern about time	.004
Concern about security	.82
Not sure how to use	.61
Uses Facebook	.24
Gender	.07

**Table 7:** Intention to use a private network for work purposes.

Factor	Beta	SE	*t*_1_	*P*	95% CI		Effect size^a^
					LL	UL	
Privacy	–0.382	0.166	–2.296	.02	–0.711	–0.052	0.046
Time	0.561	0.149	3.765	<.001	0.266	0.856	0.115
Confidence: social and computer	0.211	0.111	1.901	.06	–0.009	0.431	0.032
Age	0.008	0.010	0.763	.45	–0.012	0.028	0.005
Usefulness	0.318	0.095	3.327	.001	0.128	0.507	0.092
Basic registrar	1.371	0.346	3.963	<.001	0.685	2.056	0.126
Advanced registrar	0.998	0.390	2.558	.01	0.225	1.771	0.057
Subsequent registrar	0.884	0.346	2.550	.01	0.197	1.570	0.056
Supervisor	0.693	0.298	2.321	.02	0.101	1.284	0.047
Medical educator	0^a^						

^a^ Measured by partial eta squared.

## Discussion

### Principal Findings

The purpose of this study was to assess whether general practice registrars and supervisors in Australia would use a VCoP in the form of a private online network for training purposes and what factors are important in this decision. The results demonstrate that doctors in this sample have the access and interest needed to use a VCoP. High levels of access to computers and the Internet were coupled with overall high computer confidence. Although computer confidence was high, confidence using social media tools was lower and varied significantly between registrars and supervisors, and between applications. Confidence was also found to be related to training stage and age, but given that training stage and age are related, it was interesting to see in the regression that training stage became significant but age did not. This is in-line with previous findings that age is not a significant predictor of physicians’ use of social media [20]. Therefore, the most receptive group of doctors may be those at a more junior training stage, rather than those who are the youngest.

Confidence was found to correlate with intention to use an online community, but did not reach significance in the generalized linear regression. This may be because confidence overlaps with training stage and, thus, it is the training stage that is the greatest predictor with confidence of secondary importance. However, confidence may still be worth considering when in the implementation of a virtual community. A study from the United Kingdom showed high levels of interest in social media among British doctors, but low levels of usage, with the authors concluding training as a potential gap [21]. This suggests that a lack of training or exposure results in a lack of confidence.

In spite of good levels of access and confidence, overall use of social media for work purposes was low. This is in contrast to a recent study in the United States that showed a high uptake of social media tools, in particular physician-only communities, with 52% of respondents using online communities, such as Sermo or Ozmosis [20]. This contrast may reflect a more mature market in the United States with a longer history of online communities. In the United States, the largest online community launched in 2006 and now has more than 125,000 members, whereas in Australia serious online medical communities only began to appear in 2010.

Perceived usefulness is another important predictor of use of an online community in this study. Initially it was thought that respondents’ levels of perceived usefulness and intention to use an online community could be covariate, but this was not the case and usefulness was an independent predictor of intention to use an online community. This is in keeping with findings of 2 studies of use and intention to use social media among health care professionals, and previous studies on technology acceptance [20,22,23]. The Technology Acceptance Model was developed to describe the most significant predictors of technology use in the general community. The most significant was perceived usefulness of the technology [23]. In a US study of physician social media usage, physicians with a higher perception of usefulness of technology overcame their barriers to use [20], and in Canada, participants in a stroke knowledge transfer planning study expressed high levels of perceived usefulness of social media tools for stroke knowledge exchange [22]. The authors of the Canadian stroke study perceived a higher level of usefulness for rural users, but respondents in their study did not support this, consistent with the finding in the current study that rurality was nonsignificant. It may be that rural users are seen as the beneficiaries of online knowledge sharing tools, and this has been the case in other studies, for example, knowledge sharing among emergency medicine workers in Canada [23]. One reason for the difference may be that general practice registrars can experience structural isolation as a result of working in small practices with less professional contact than hospital workers, in urban as well as rural environments [1]; conversely, as in the Canadian stroke study, respondents may already have strong established local networks [22]. Perceived usefulness is also important because it is potentially modifiable through training and promotion of the potential benefits of an online community to its users.

Finally, barriers are important to address. In this study, time and concerns about privacy were important negative predictors of use, but concerns about security were not significant. This may have been because of a lack of understanding of the difference between privacy and security, or a lack of concern about security, or a higher value being placed on personal or patient confidentiality than computer security. In contrast to these possible concerns, in the Canadian stroke study, participants did not express particular concern regarding patient confidentiality in online exchanges [22]. Once again, this may be due to a more evolved North American market with more experience in online exchanges, as the participants were said to be “fully aware that written communication within a Web platform must ensure confidentiality and respect ethics rules” [22]. Time as a barrier correlates with the findings of the recent Canadian stroke study [22], and a number of previous studies on health professional use of VCoPs [24,25]. It is a difficult factor to modify. However the US physician Web 2.0 study found that in spite of a high perception of barriers, if usefulness and ease of use are taken into account, usage is still high [20]. Thus, the barrier of time needs to be recognized and addressed with training and promotion on potential usefulness.

Ease of use of a network is another important consideration [23]. The preference among doctors for a private network compared with an open network for work and training purposes was significant and most likely related to privacy concerns. This is supported by their lack of preference for a private network when using an online network for social purposes in which patient confidentiality is not an issue. Importantly, previous work has expressed concern that private networks may have an effect on decreasing ease of use by introducing the need for passwords [22]. Given the importance among respondents of a private network, ease of use may be able to be addressed through technical and training avenues, such as the use of a current password (ie, integrating the network with a current training platform), the ability to “remember me,” and easy retrieval of lost passwords.

The findings from this study can be looked at in terms of the proposed Health VCoP framework presented in the recent literature review of VCoPs in general practice training [8]. In that framework, elements of Probst and Borzillo’s [9] recognized business VCoP framework were modified for the health sector based on the current literature. The framework consists of 7 factors (see Textbox 1), including facilitation, champion and support, objectives and goals, a broad church, a supportive environment, measurement benchmarking and feedback, technology, and community. In the current study, in the broad church category, it seems that not only does a network need to engage users with varying abilities (eg, registrars and supervisors), registrars may actually be more likely to engage than general practice supervisors. In the technology and community category, training is an important factor when implementing a VCoP. As well as focusing on technical training, training could include promoting usefulness and confidence in using the online network, as well as addressing the barriers of time and privacy. This is consistent with findings from a US physician study in which barriers were perceived, but they were overcome if usefulness was perceived to be high [20]. This promotion of usefulness may also be a role for the facilitator. Facilitators can make sure that users are engaged, are realizing the potential of the site, that feedback is responded to, and that necessary changes are made to the site in response to feedback and usage. A facilitator can also grow the community by monitoring and ensuring the usefulness of the site for both active and passive users, as the health framework proposes that both groups are valuable to the community. Finally, if a general practice training network were to be considered, concerns about privacy would need to be addressed through design (eg, password authentication). The resulting usage barrier would need to be offset by appropriate design to ensure ease of access on the password-protected site.

**Health virtual community of practice framework based on Barnett et al [8].**

1. Facilitation

Facilitators promote engagement and maintain community standards

2. Champion and support

The network needs to have an initial stakeholder champion, with stakeholder support

3. Objectives and goals

Clear objectives provide members with responsibilities and motivates them to contribute more actively

4. A broad church

Consider involving different overlapping, but not competing, professional groups, different organizations, and external experts. However, make sure the church is not too broad

5. Supportive environment

Health VCoPs should promote a supportive and positive culture that is both safe for members and encouraging of participation

6. Measurement, benchmarking, and feedback

Health VCoPs should consider measurement as a factor in their design, including benchmarking and feedback

7. Technology and community

Online CoPs should ensure ease of use and access, along with asynchronous communication. Other options including chat and meetings can also be considered, along with the need for training
Communities are more likely to share knowledge when there is a mixture of online and face-to-face meetings, members self-select, and both passive and active users are encouraged

### Limitations

There are a number of limitations in this study. One limitation is that users self-selected to answer a survey on computing and social media by clicking a link in an email to an online survey. The resulting self-selection bias may therefore overreport computer confidence across the whole general practice registrar and supervisor population in the chosen training region. However, it should be noted that the levels of user confidence reported in this study are in keeping with, if not lower, than that found in other recent research [20]. Another limitation is that the response rate was much higher among registrars than supervisors, which may make the results for supervisor responses potentially less representative. Further research on the attitudes of supervisors is needed.

### Conclusions

General practice training can be isolating in Australia. Registrars move from a hospital environment with many colleagues, often in large urban centers, to small practices in urban and rural areas with fewer colleagues. The resulting structural, professional, and social isolation is one of the problems that can lead registrars to consider reducing working hours and moving away from rural work. The Australian general practice workforce is already under pressure, and if isolation can be addressed, this has positive implications for quality of primary care delivery and retention of a rural workforce.

Virtual communities of practice are an effective means of overcoming professional isolation in the business sector and show promise in the health sector. They can overcome isolation by providing a vehicle for knowledge sharing and social interaction. This study shows that general practice registrars and supervisors, in particular registrars, have the access, confidence, and interest to use a VCoP for work and training purposes. The main drivers for use appear to be perceived usefulness and a more junior training stage, with a suggestion that current computer and social media confidence is also beneficial. Barriers to use such networks include time and privacy.

These findings fit with some of the aspects of the Barnett et al [8] health VCoP framework (see Textbox 1). In particular, they provide some pointers for implementing a VCoP for general practice training. Given their high interest and confidence, general practice registrars may be the easiest group with which to pilot such a network. In doing so, consideration needs to be given to design, maximizing ease of use, while barriers around time and privacy are addressed through training and promotion. Lastly, despite some apparent barriers, if adequate consideration is given to promotion and training to demonstrate usefulness, these barriers may well be overcome.

## Abbreviations

CoP: community of practice
VCoP: virtual community of practice
